# Acute mesenteric ischemia secondary to superior mesenteric vein thrombosis in a patient with liver cirrhosis: A case report

**DOI:** 10.1097/MD.0000000000034549

**Published:** 2023-08-11

**Authors:** Stefan Mitev, Antoniya Topalova-Dimitrova, Anton Varlyakov, Dimitar Popov

**Affiliations:** a Gastroenterology Clinic, University Hospital Sv Ivan Rilski, Sofia, Bulgaria; b Radiology Department, University Hospital Sv Ivan Rilski, Sofia, Bulgaria.

**Keywords:** acute mesenteric ischemia, liver cirrhosis, superior mesenteric vein thrombosis

## Abstract

Acute mesenteric ischemia due to superior mesenteric vein (SMV) thrombosis is a rare yet potentially life-threatening emergency. Our case report explores this condition in the context of a patient with liver cirrhosis due to Wilson disease. We specifically highlight the complex derangement of the coagulative balance in liver cirrhosis.

**Patient concerns:**

A 34-year-old female with Wilson disease-related cirrhosis presented with intractable abdominal pain, nausea, and vomiting that showed no response to antispasmodic medication.

**Diagnoses:**

A contrast-enhanced abdominal computed tomography scan and Doppler ultrasound confirmed an intraluminal filling defect in the SMV, leading to the diagnosis of SMV thrombosis.

**Interventions:**

Prompt anticoagulation, intravenous fluids, and an antibiotic were initiated. Surgical consultation recommended conservative therapy with close monitoring.

**Outcomes:**

Over the following 2 days, the patient’s condition improved considerably, with almost complete resolution of her symptoms. Genetic testing identified a 4G/4G homozygous genotype of the plasminogen activator inhibitor 1 gene, associated with a higher risk of thrombosis in the vessels of internal organs. After 2 months of sustained anticoagulant therapy, a follow-up contrast-enhanced computed tomography scan revealed near-complete recanalization of the SMV, and the patient remained symptom-free.

**Lessons:**

This case underscores the importance of early detection and treatment of acute mesenteric ischemia in patients with liver cirrhosis, as well as the potential role of genetic factors in thrombosis.

## 1. Introduction

The superior mesenteric vein (SMV) drains the embryological midgut and combines with the splenic vein to form the portal vein. Acute SMV thrombosis is a rare yet life-threatening cause of intestinal ischemia. Early diagnosis through imaging and timely initiation of anticoagulant therapy are essential for preventing complications and improving outcomes. We present a case of SMV thrombosis occurring in a patient with liver cirrhosis and a genetic mutation associated with a higher risk of thrombosis in the vessels of internal organs.

## 2. Case presentation

A 34-year-old female presented with intractable abdominal pain, nausea, and vomiting, which began 1 day prior. She was diagnosed with cirrhosis due to Wilson disease 7 years ago and was on maintenance therapy with D-penicillamine. Her most recent urinary copper excretion test was within the target range. The patient denied hematemesis, hematochezia, dyspnea, fever, alcohol use, smoking, allergies, or recent travel history. She reported no relief from antispasmodic medication and denied experiencing similar symptoms in the past.

**Figure 1.**
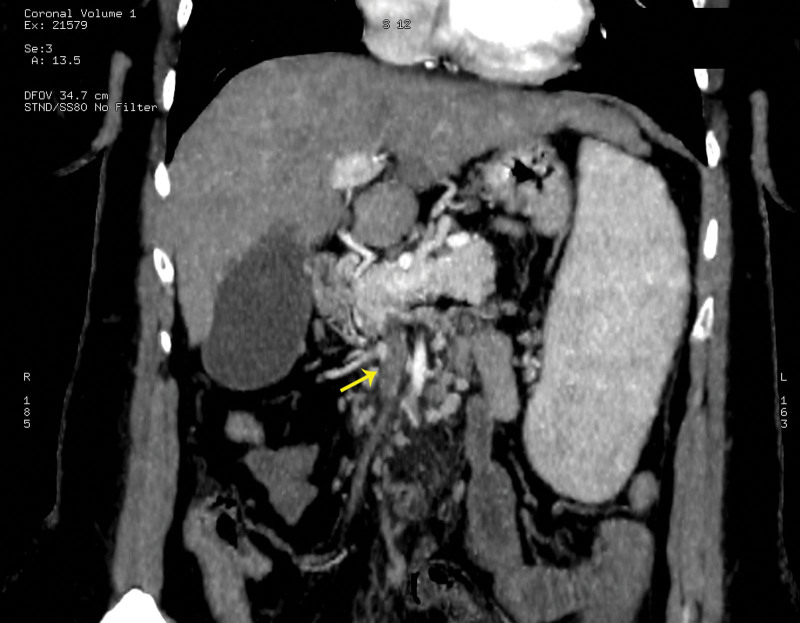
Intraluminal filling defect (yellow arrow) in the superior mesenteric vein on contrast-enhanced CT scan. CT = computed tomography.

**Figure 2.**
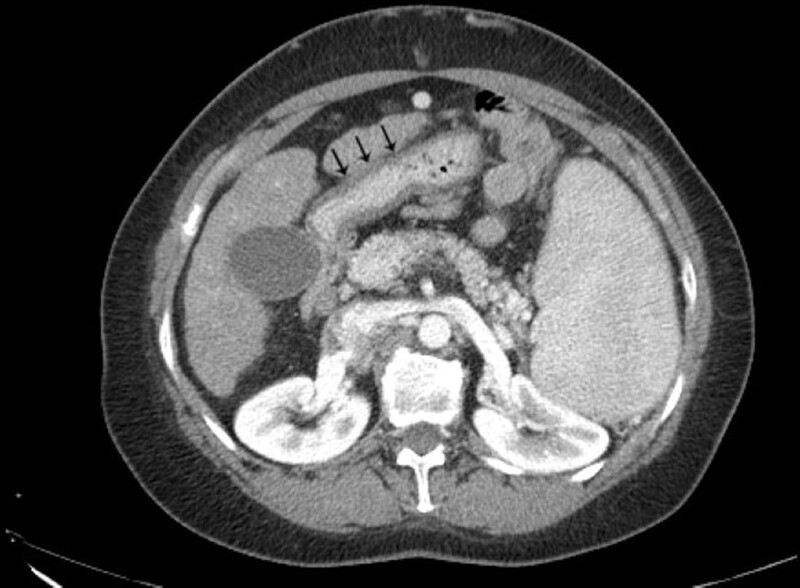
Duodenal wall thickening (black arrows) due to ischemia on contrast-enhanced CT scan. CT = computed tomography.

On physical examination, the patient was found to be hypotensive with a blood pressure of 90/60 mm Hg and a pulse rate of 88 beats per minute. Abdominal examination was significant for tenderness on palpation in the epigastrium, without rebound or guarding. Initial laboratory investigations were notable for low platelet count of 67 × 10^9^/L (normal range 130–360 × 10^9^/L), elevated C-reactive protein of 14.2 mg/L (normal value < 6 mg/L), and International Normalized Ratio of 1.21 (normal range 0.9–1.2). Hemoglobin, white blood cell count, total and direct bilirubin, serum albumin, aspartate aminotransferase, alanine aminotransferase, alkaline phosphatase, and amylase were all within normal limits. Upon admission, Model For End-Stage Liver Disease score was 9 and Child-Turcotte-Pugh class was A. An urgent contrast-enhanced abdominal computed tomography (CT) scan was performed, and representative images are shown in Figures 1 and 2.

The abdominal CT scan revealed an intraluminal filling defect in the SMV, as well as duodenal wall thickening. Liver cirrhosis and splenomegaly were also noted. Transabdominal color Doppler ultrasound confirmed the complete absence of blood flow in the SMV. Based on these findings, a diagnosis of acute mesenteric ischemia due to SMV thrombosis was made.

Anticoagulant therapy with low-molecular-weight heparin (LMWH) (nadroparin calcium 5700 IU anti-Xa/0.6 mL b.i.d.) was promptly initiated. The patient received intravenous fluids and an antibiotic (ceftriaxone). The results for anti-cardiolipin antibodies, anti-Beta2 glycoprotein I antibodies, anti-prothrombin antibodies, and anti-annexin V antibodies were unremarkable. The D-dimer level was elevated at 7.35 mg/L (normal value < 0.55 mg/L). Surgical consultation recommended to proceed with conservative therapy with close monitoring. Over the following 2 days, the patient’s condition improved considerably, with almost complete resolution of her symptoms.

Esophagogastroduodenoscopy prior to discharge revealed tortuous esophageal varices without red wale marks. The duodenal mucosa appeared edematous and friable, showing submucosal hemorrhages but no signs of necrosis. Further genetic testing identified a 4G/4G homozygous genotype of the plasminogen activator inhibitor 1 gene.

**Figure 3.**
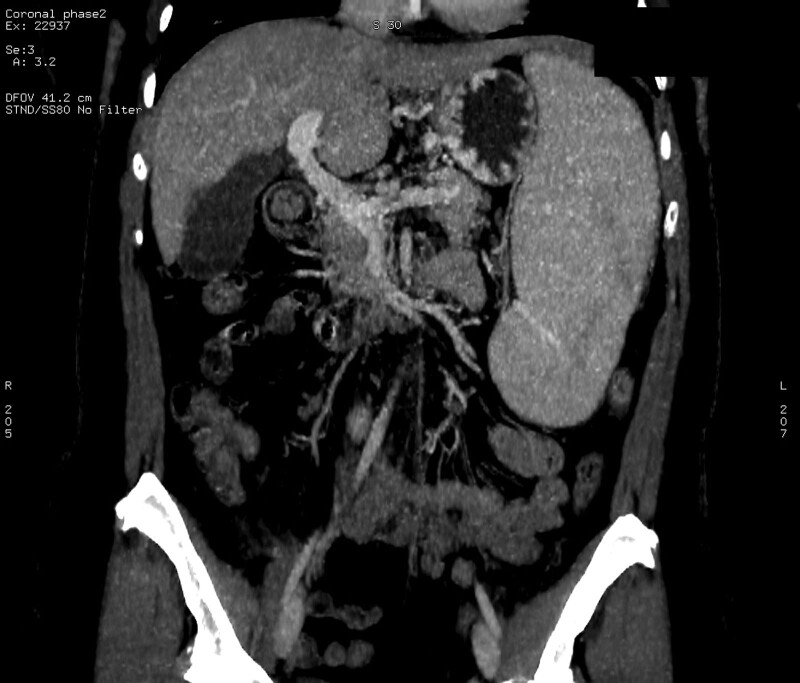
Near-complete recanalization of the superior mesenteric vein on follow-up contrast-enhanced CT scan 2 months later. CT = computed tomography.

After a 10-day hospital stay, the patient was discharged without symptoms and remained on LMWH anticoagulant therapy (nadroparin calcium 3800 IU anti-Xa/0.4 mL b.i.d.) and a nonselective beta-blocker to prevent variceal bleeding. Two months later, a follow-up contrast-enhanced CT scan revealed near-complete recanalization of the SMV (Fig. 3). Additionally, the D-dimer levels gradually decreased until they reached normal levels. The dosage of nadroparin calcium was reduced to 3800 IU anti-Xa/0.4 mL q.d. with potential consideration for a future switch to a direct oral anticoagulant. The patient will be followed up with transabdominal Doppler ultrasound every 3 months during the first year.

## 3. Discussion

Acute thrombosis of the SMV, initially described in 1894, is a rare cause of intestinal ischemia, with an incidence of 1.8 per 100,000 person-years.^[1]^ Potential causes of thrombophilia or hypercoagulable state may include myeloproliferative disease, antiphospholipid syndrome, factor V Leiden mutation, paroxysmal nocturnal hemoglobinuria, hereditary deficiencies of protein S, protein C, and antithrombin, hyperhomocysteinemia, and oral contraceptive use. Thrombotic occlusion in the SMV leads to inadequate perfusion pressure and stagnating flow, resulting in significant bowel wall edema. This can progress to submucosal hemorrhage, ischemia, and ultimately complete bowel infarction. Patients with SMV thrombosis may present with nonspecific and variable symptoms, such as nausea, vomiting, diarrhea, and abdominal pain that is disproportionate to physical findings. CT angiography and magnetic resonance angiography are the preferred imaging modalities due their ability to provide clear visualization of the mesenteric veins and detect signs of intestinal infarction. Doppler ultrasonography can be used as an initial noninvasive method, although it may have lower sensitivity.^[2]^ Prompt initiation of treatment in cases of acute SMV thrombosis is crucial to prevent complications. Urgent surgical intervention is indicated for patients suspected to have bowel gangrene or perforation. Mesenteric vein thrombosis is associated with a 30-day mortality rate of 20%, primarily due to bowel infarction.^[3]^ The optimal type and dose of anticoagulant therapy for splanchnic venous thrombosis remain uncertain, as most evidence is primarily based on observational studies.^[4]^ In cases of reversible etiology, it is recommended to continue anticoagulation for 3 to 6 months, whereas for underlying thrombophilia, anticoagulation should be indefinite.^[5]^ Meta-analyses have shown comparable low bleeding risk for vitamin K antagonists, LMWH, and direct oral anticoagulants. Consistently, anticoagulation has been associated with lower rates of recurrent thrombosis, major bleeding, and mortality.^[6]^

It is important to note that patients with liver cirrhosis who present with a low platelet count and prolonged prothrombin time should not be considered “automatically anticoagulated” and therefore protected from thrombotic events. Their coagulation status is more complex, involving alterations in both procoagulant and anticoagulant factors. The risk of thrombus formation, especially in the portal vein, may be increased due to reduced anticoagulant pathway activity, slow blood flow caused by stasis, and abnormal fibrinolysis.^[7]^ Conversely, bleeding in these patients is more closely related to the degree of portal hypertension rather than hemostatic imbalances. Furthermore, our patient exhibited the 4G/4G genotype of the plasminogen activator inhibitor 1 gene, which has been associated with a higher risk of thrombosis in vessels of internal organs.^[8]^ This mutation leads to higher levels of plasminogen activator inhibitor 1, a protein that inhibits fibrinolysis. In patients with liver cirrhosis, this could potentially further exacerbate the imbalance in the coagulation system.

## 4. Conclusion

Acute thrombosis of the SMV is a rare cause of intestinal ischemia. Early initiation of treatment is crucial to prevent complications, and the optimal type and dose of anticoagulant therapy remain uncertain. Patients with liver cirrhosis presenting with a low platelet count and prolonged prothrombin time should not be considered “automatically anticoagulated.” Their coagulation status is complex, and the risk of thrombus formation may be increased.

## Acknowledgments

We would like to express our gratitude to the patient for allowing us to share this case.

## Author contributions

**Conceptualization:** Stefan Mitev, Antoniya Topalova-Dimitrova.

**Data curation:** Antoniya Topalova-Dimitrova.

**Investigation:** Anton Varlyakov.

**Supervision:** Dimitar Popov.

**Visualization:** Anton Varlyakov.
